# Carbon dots as fluorescent probes for detection of VB_12_ based on the inner filter effect†

**DOI:** 10.1039/c8ra03070g

**Published:** 2018-05-29

**Authors:** X. Y. Sun, M. J. Yuan, B. Liu, J. S. Shen

**Affiliations:** College of Materials Science and Engineering, Huaqiao University, Key Laboratory of Molecular Designing and Green Conversions (Fujian University) Xiamen 361021 China sunxy@hqu.edu.cn +86-592-6160088 +86-592-6162231

## Abstract

In this study, we constructed a new fluorescent sensing for VB_12_ and investigated the mechanism of vitamin B_12_ (VB_12_) quenching fluorescence of carbon dots (CDs). The fluorescence suppression is attributed to the inner filter effect (IFE) because of the overlap between UV-vis absorption spectrum of VB_12_ and emission/excitation spectra of CDs. This CDs-based sensor provides obvious advantages of simplicity, convenience, rapid response, high selectivity and sensitivity, which has potential application for the detection of VB_12_ in the medical and food industry.

## Abstract 2

We constructed a new fluorescent sensing for vitamin B_12_ (VB_12_) and investigated the mechanism of VB_12_ quenching fluorescence of carbon dots.

## Graphical abstract

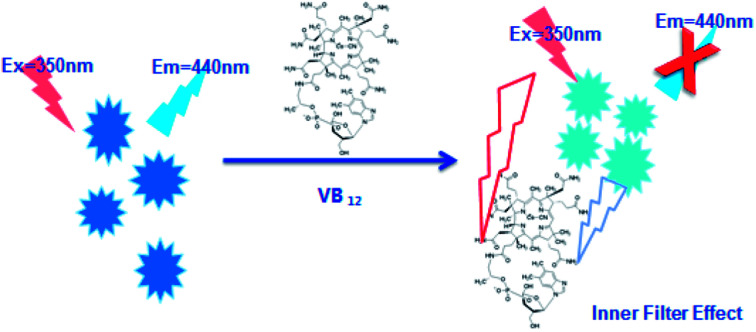

## Introduction

Vitamin B_12_ (VB_12_), also called cobalamin, is an essential water soluble nutrient that can be found in foods such as meat, eggs and dairy products. It can effectively prevent diseases such as pernicious anemia, senile dementia, depression. Therefore, VB_12_ plays an integral role in keeping the human body healthy. However, excess VB_12_ can also produce toxic side effects, such as a lack of folic acid. So, the detection of VB_12_ is very important.

In the past years, numerous analytical methods for the determination of VB_12_ in different sample matrices have been successfully developed, including microorganism method, HPLC-UV, atomic absorption spectroscopy, thin layer chromatography, *etc.* However, most of these methods are not perfect due to the requirement of expensive equipment, being time consuming or complicated sample pretreatment. Therefore, a simple, sensitive, and selective method for VB_12_ detection is highly demanded. Fluorescence analysis has been applied in the detection of VB_12_ due to its high sensitivity and easy operation. In 2014, Kolekar's group^1^ detected VB_12_ based on FRET between CdS and VB_12_, and this method could be used in serum and urine actual samples. Other groups used rhodamine B^2^ or gold nanoclusters modified by bovine serum protein^3^ as fluorescent probes to detect VB_12_. However, these methods had the following problems: the fluorescent probes had biological toxicity, the operations were complex and the sensitivities were not high. So choosing a green fluorescent probe and a simple operation can help constructing a better fluorescent method to detect VB_12_.

Compared with traditional quantum dots and fluorescent dyes, carbon dots (CDs) have a series of unique properties such as excellent water solubility, anti-photobleaching, easy of modification, low toxicity, low cost, tunable excitation and emission spectra.^4–7^ These attractive features indicate the prominent advantages of CDs in chemical sensing, biosensing, bioimaging, nanomedicine and catalyst. CDs have been applicated in the sensing of ions (such as Hg^2+^, Cu^2+^, Fe^3+^, F^−^ and BrO_3_^−^, *etc.*),^8–12^ molecular substances (for instance, glucose,^13^ hemoglobin,^14^*p*-nitrophenol^15^*etc.*) and pH.^16^ The mechanisms of CDs-based fluorescence sensor include photo induced electron transfer (PET), intramolecular charge transfer (ICT), fluorescence resonance energy transfer (FRET) and twisted intramolecular charge transfer (TICT), *etc.* Inner filter effect (IFE) is also a mechanism of fluorescence sensing. The IFE refers that the excitation and emission spectra of fluorophores are absorbed by absorbers, then the fluorophores suppress.^17^ In the beginning, IFE was an inevitable error, the research on the IFE mainly focused on the correction of IFE. Since metal nanoparticles have good absorption coefficient, they can be used as good absorbers.^18^ Now, the IFE has been developed into an analytical method.^19–24^ Fluorescence detection based on IFE includes two forms. IFE can happen between metal nanoparticles and other fluorophores, and an analyte can make fluorescence recovery. IFE can also happen between an analyte and fluorophores, and the analyte can be detected based on fluorescence quenching. In this study, VB_12_ was detected based on the IFE between VB_12_ and CDs. This method has an extremely low LOD for detecting VB_12_ of 93 nM, and the detection system is simple and fast.

## Experimental

### Reagents and equipments

Ammonium citrate (AR) and all the coexisting vitamins were obtained from Aladdin Chemistry Company Limited (Shanghai, China). Vitamin B_12_ (VB_12_) was purchased from Beijing Fangcao Medicine Chemical Industry Developed Company. NaH_2_PO_4_·2H_2_O, Na_2_HPO_4_·12H_2_O, NaOH, H_3_PO_4_, H_3_BO_3_, CH_3_COOH and all the coexisting ions were obtained from Guoyao Company (Shanghai, China). VB_12_ tablets were purchased from Yunpeng Medical Company (Shanxi, China) and VB_12_ injections were obtained from Ruicheng Tiantong Company Limited (Shanxi, China). All chemicals were used in the experiments without further purification. Deionized water, purified by Millipore system (18.0 MΩ cm at 25 °C), were employed for all experiments.

pH was measured by a Model 1828 digital pH meter. PL spectra were acquired by a Hitachi F-7000 spectrometer. UV-vis absorption spectra were recorded by a Shimadzu UV-2600 spectrophotometer. FTIR-4800S spectra were employed for obtaining IR spectra in KBr discs in the 4000–400 cm^−1^ region. X-Ray Diffraction (XRD) results were recorded on a Rigaku Smart lab with a speed of 6° per minute. Transmission electron microscopy (TEM) experiments were done on a TECNAI-F30 system. Zeta potential and dynamic light scattering (DLS) size distribution were obtained by a Zetasizer Nano.

### Preparation of CDs

The fluorescent CDs were prepared through a simple and low-cost hydrothermal treatment.^19^ Briefly, 2.0 g of ammonium citrate was added into 25 mL distilled water. The solution was heated from room temperature to 160 °C in a 50 mL *para* polyphenol (PPL) equipped stainless steel autoclave and held at 160 °C for 6 h. The color of the solution gradually turned into deep blue from colorless in appearance. When the resulting solution was cooled to room temperature, the solution was placed in the fridge for further application. The product can be used directly without any further passivation or purification.

### Vitamin B_12_ sensing

For sensing vitamin B_12_ (VB_12_), 1.2 μM CDs solution and 2 mL 0.2 M PB buffer solution of pH 7.0 were mixed with the solution containing VB_12_ of various concentration to afford a fixed volume of 5 mL. After stirring, the mixed solution was maintained at room temperature for 15 min, PL spectra were measured. Moreover, the experiments of coexistence of vitamins or heavy metal ions were conducted for further investigating the selectivity under similar experimental conditions of this sensing system. The excitation wavelength was set as 350 nm.

### Interference experiments of VB_12_

To investigate the effects of other coexisting substances to VB_12_ detection, some vitamins (VB_1_, VB_3_, VB_5_, VB_7_, VB_9_, VC) and some metal ions (Ba^2+^, Ca^2+^, Cd^2+^, Co^2+^, Cr^3+^, Cu^2+^, Zn^2+^, Ni^2+^, Mn^2+^, Fe^2+^, Fe^3+^, Hg^2+^, Pb^2+^) were added to CDs solution with VB_12_. The concentration of Hg^2+^ was 10 μM, Fe^2+^ and Fe^3+^ were 40 μM. Then vitamins and other metal ions were 100 μM.

## Results and discussion

### Synthesis and characterization of CDs

The CDs with blue fluorescent were prepared by a hydrothermal treatment. The optical properties of the CDs are shown in Fig. S1.† As shown in the UV-vis absorption spectra, one absorption peak was at 234 nm, due to π → π* transition of C=C bond,^20^ and another peak at 338 nm was attributed to n → π* transition of C=O bond.^21^ The photoluminescence (PL) spectra of CDs showed that the maximum peak centered at 440 nm under a 350 nm excitation wavelength, which presented a bright blue color under UV lamp. And the emission spectra didn't shift red with the excitation changing.

**Fig. 1:**
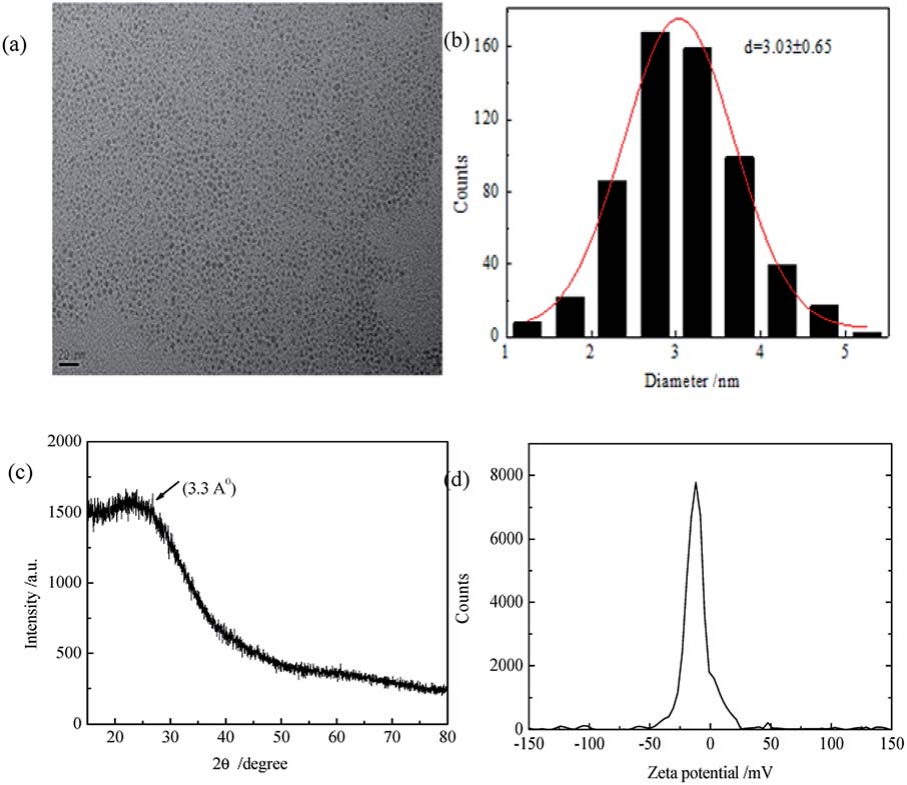
(a) TEM image of CDs; (b) particle size distribution of CDs; (c) XRD pattern of CDs (d) zeta potential of CDs.

The QY of CDs was 0.24 by calibrating against reference quinine sulfate in H_2_SO_4_ and the lifetime was 6.52 ns fitting with single index. From Fig. 1, we found that the as-prepared CDs had an average size of 3.03 nm and they were mono-dispersed. XRD showed the CDs had 002 facet which means graphene layers. The FTIR spectrum showed the surface of CDs exist amide functional, hydroxyl groups and carboxyl groups. Zeta potential results showed a value of −11.73 mV, probably resulted from the slight ionization of hydroxylgroup of the surface of the resulting CDs. DLS measurement revealed that the CDs particles had good size distribution with an average size of 9.68 nm. The synthesized CDs had good light stability, as showed in Fig. S2.†

### The optimization of important factors for the probe

**Fig. 2:**
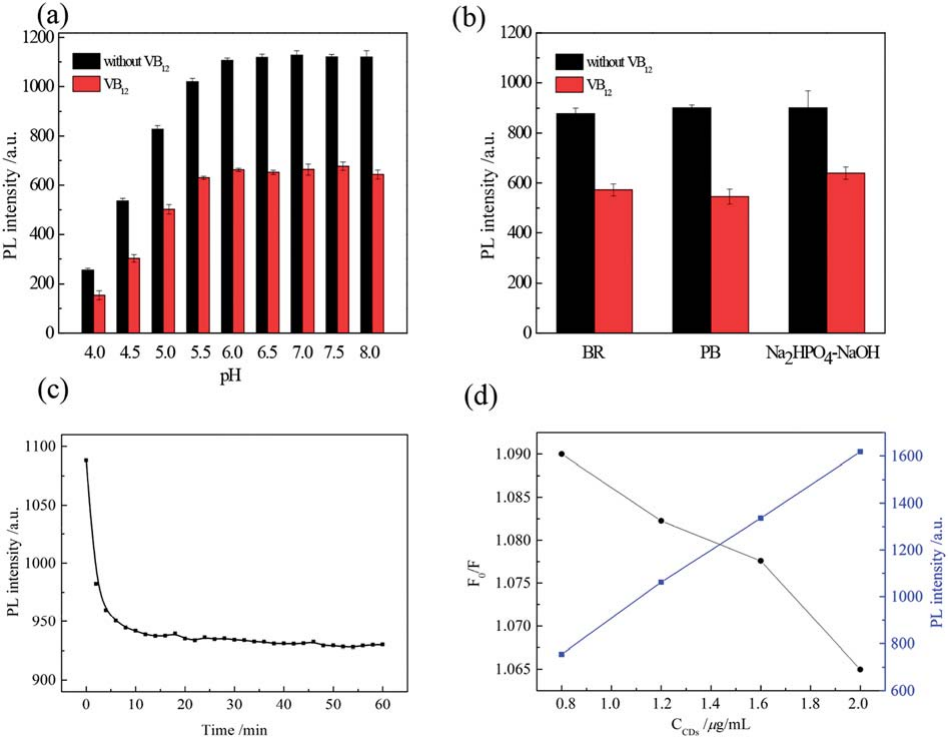
(a) PL intensity of CDs in the absence (black) and presence (red) of 15 μM VB_12_ in BR buffer solution of various pHs; (b) PL intensity of CDs in the absence (black) and presence (red) of 15 μM VB_12_ in different buffer solutions; (c) time-dependent fluorescence intensity of CDs to 15 μM VB_12_; (d) effect of CDs concentration on fluorescence quenching.

The effects of pH on the PL of CDs were investigated. As shown in Fig. 2(a), under acidic pH, the PL intensity of CDs decreased. This could be attributed to the carboxyl groups on the CDs surface accumulated by combining the protons. The PL intensity and the quenching kept almost constant in the pH range of 6.0–8.0. In this experiment, we selected pH = 7.0. Types of buffer were also investigation. In PB buffer, the most quenching was obtained as shown in Fig. 2(b). Then the reaction was fully completed within 15 min, as shown in Fig. 2(c), suggesting a rapid method to detect VB_12_. The CDs concentration was investigated and the optimal concentration was 1.2 μM.

### Establishment of the sensing system for VB_12_

**Fig. 3:**
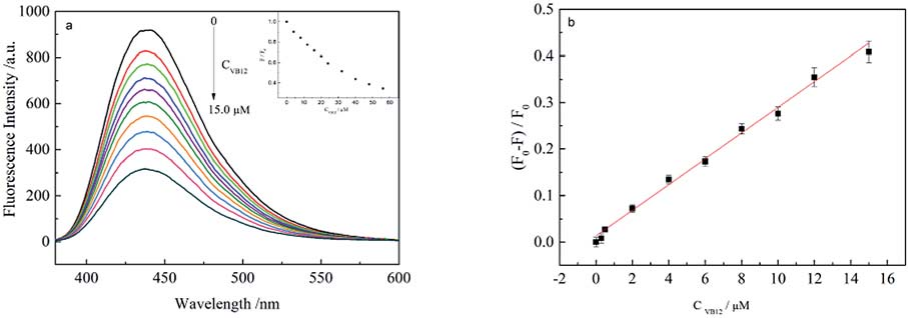
(a) PL spectra of CDs in VB_12_ with different concentrations; (b) the relationship curve between (*F*_0_ − *F*)/*F*_0_ and VB_12_ concentration.

Fig. 3(a) shows the PL response of the CDs towards VB_12_. It was found that the PL intensity of CDs gradually decreased with increasing VB_12_ concentrations (0, 0.3, 0.5, 2.0, 4.0, 6.0, 8.0, 10.0, 12.0, 15.0 μM). Fig. 3(b) shows a linear correlation curve could be fitted between VB_12_ concentration and (*F*_0_ − *F*)/*F*_0_. In which *F*_0_ is the PL intensity of CDs without VB_12_ and *F* with different VB_12_ concentrations. A good linear range was within 0.3–15 μM and the linear correlation coefficient could be obtained to be 0.9940. The limit of detection (LOD) was also calculated to be 93 nM, according to the equation of LOD = 3*σ*/*k*, in which *σ* is the standard deviation from 11 blank solutions and *k* is the linear slope fitted. Compared with other methods of fluorescence detection of vitamin B_12_, this method is green environment and less pollution. The detection system is simple and fast, the linear range is wide and the sensitivity is high, as shown in Table 1.

**Table 1:** Comparison of our proposed method and the reported cases to detect VB_12_ based on PL

Sensor	Linear range	LOD	Ref.
Graphene oxide nanolayer	0–1.08 μM	320 nM	1 (ref. 18)
CdS	3.7–73.8 μg mL^−1^	5.1 μM	2 (ref. 19)
CdTe	0.7–1.8 μM	0.1 μM	3 (ref. 20)
CDs	0.3–15 μM	93 nM	This work

### Effect of coexisting substances in the determination of VB_12_

**Fig. 4:**
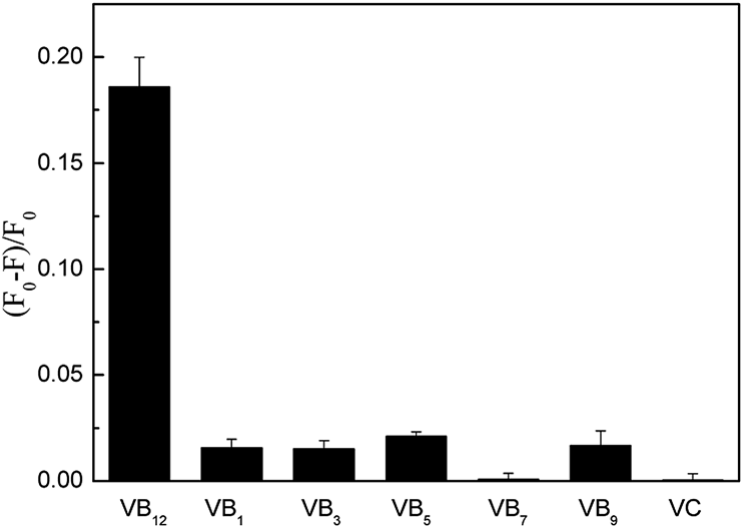
Selectivity of CDs for VB_12_ over other vitamins (VB_12_ concentration was 8 μM. VB_9_ was 10 μM and other vitamins were 100 μM).

In order to explore the possibility of practical application in the determination of VB_12_, the interferences from other vitamins and anions were tested under the optimized conditions. Fig. 4 showed other vitamins have no influence to the detection of VB_12_. And Table 2 showed that some anions quenched the fluorescence of CDs, such as Hg^2+^. From Fig. S3,† the coexisting Hg^2+^ could suppress the PL intensity of CDs heavily while the effect of Hg^2+^ coexisting could be eliminated by adding EDTA.

**Table 2:** Effect of metal irons (8 μM) on the detection of VB_12_

Coexisting substance	Concentration (μmol L^−1^)	Change of fluorescence intensity (%)
Ba^2+^	100	+0.01
Ca^2+^	100	+3.50
Cd^2+^	100	+3.07
Co^2+^	100	+5.13
Cr^3+^	100	+2.42
Pb^2+^	100	−5.31
Cu^2+^	100	+0.77
Zn^2+^	100	+0.80
Ni^2+^	100	+2.09
Mn^2+^	100	+3.60
Fe^2+^	40	+2.81
Fe^3+^	40	+2.87
Hg^2+^	10	−73.60

### Fluorescence quenching mechanism of CDs by VB_12_

**Fig. 5:**
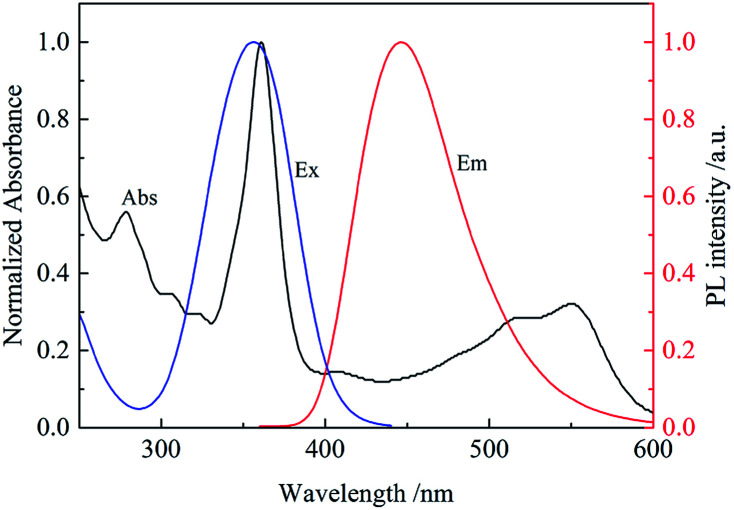
Normalized UV-vis absorption spectrum of VB_12_, excitation and emission spectra of CDs.

**Fig. 6:**
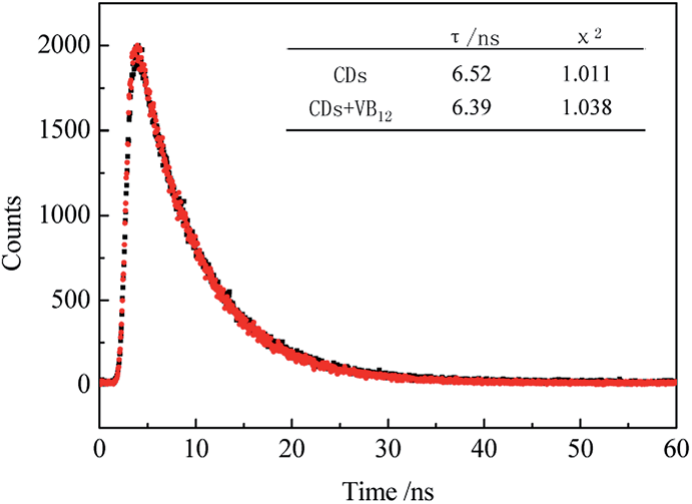
Lifetime of CDs in the absence (black) and presence (red) of 4 μM VB_12_.

**Fig. 7:**
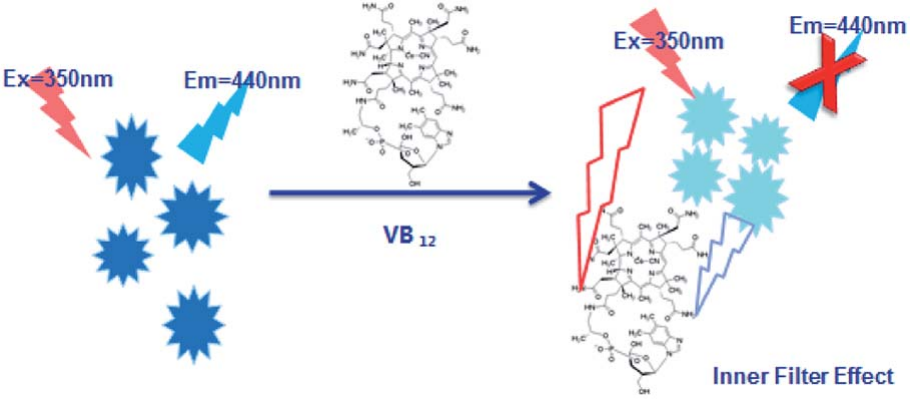
Schematic illustration of the detection of VB_12_ by CDs.

**Fig. 8:**
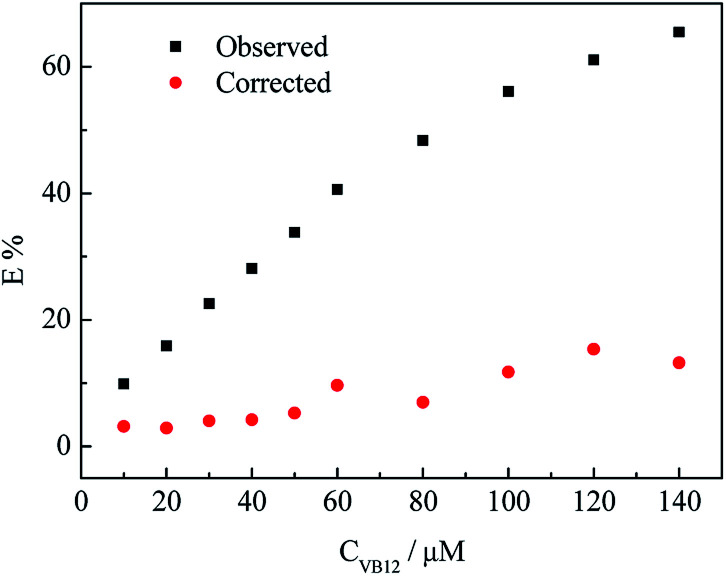
Suppressed efficiency (*E*%) of observed (black) and corrected (red) measurements for CDs after each addition of different concentrations of VB_12_.

It was found that the fluorescence of CDs could be quenched by VB_12_, revealing the possibility of applying the as-prepared CDs as a sensitive fluorescent sensor of VB_12_. From the spectra in Fig. 5, we could see a good spectral overlap between the absorption spectrum of VB_12_ and the excitation and emission spectra of CDs, suggesting that the fluorescence quenching might be related to FRET or IFE. Since lifetimes of CDs both in the absence and presence of VB_12_ remained unchanged (Fig. 6), fluorescence quenching was unreasonable ascribed to the FRET process. Then there wasn't blue shift or red shift of CDs emission which shows no interaction appeared between CDs and VB_12_. Furthermore, the selectivity toward VB_12_ could be explained by the IFE mechanism (Fig. 7). The IFE can be estimated according to the following equation.^25^1
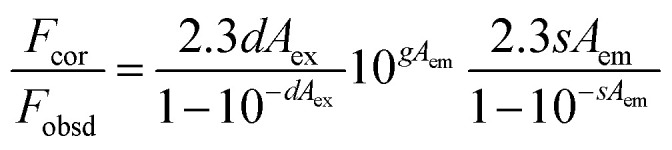
wherein, *F*_obsd_ is the measured maximum fluorescence intensity and *F*_cor_ is the corrected maximum fluorescence intensity by removing IFE from *F*_obsd_. *A*_ex_ and *A*_em_ represent the absorbance at the excitation wavelength (*λ*_ex_ = 350 nm) and maximum emission wavelength (*λ*_em_ = 446 nm), respectively; *s* is the thickness of excitation beam (0.10 cm^26,27^); *g* is the distance between the edge of the excitation beam and the edge of the cuvette (0.40 cm) and *d* is the width of the cuvette (1.00 cm). In Table S1,† CF (correction factor) was that *F*_cor_/*F*_obsd_, which could be calculated by eqn (1.1). In order to ensure the credibility of the correction, the maximum value of CF could not exceed 3. *F*_cor_ was the PL intensity of CDs with different VB_12_ concentrations after IFE correction and it could be calculated by eqn (1). *F*_cor,o_ was the PL intensity of CDs without VB_12_ after IFE correction and in Table S1† it was 964.95. *F*_cor,o_/*F*_cor_ could reflect the degree of quenching after IFE correction. The closer the ratio was to 1, the greater the effect of the IFE. *E*% (*E* = 1 − *F*/*F*_0_) was suppressed efficiency, *F*_0_ and *F* were the FL intensities of CDs in the absence and presence of VB_12_, respectively. In Fig. 8, we found that approximately all of the suppressed effect came from the IFE of VB_12_. After removing the IFE, there was almost no quenching in linear range. Therefore, approximately all of the quench effects come from the IFE of VB_12_.

The correction factor at each concentration of VB_12_ thus could be calculated (Table S1†), *F*_cor,o_. is the corrected fluorescence intensity in the absence of VB_12_. After the IFE was removed from the totally observed suppressed fluorescence, the suppressed efficiency *E* = 1 − *F*/*F*_0_, for the totally observed and the corrected fluorescence of VB_12_ is figured out, as shown in Fig. 8. We found that approximately all of the quench effects come from the IFE of VB_12_.

### Practical application

The method in real application was also investigated in the determination of VB_12_ in injections and tablets samples. VB_12_ samples of various known concentrations were employed to conduct the recovery experiments. The experimental results were summarized in Table 3. The recoveries ranging from 93.3% to 109.2% were acceptable which indicated our method had good reliability of the sensing system.

**Table 3:** Detection of VB_12_ in real samples (*n* = 3)

Samples	Added (μM)	Spiked (μM)	Found (μM)	Recovery (%)	RSD (%)
Injections	1.8	3.0	4.7	96.7	1.27
	1.8	8.0	9.6	97.5	1.19
	1.8	13.0	14.2	95.4	0.67
Tablets	1.8	3.0	4.6	93.3	0.96
	1.8	8.0	9.7	98.8	0.51
	1.8	13.0	16.0	109.2	1.62

## Conclusions

In this paper, a fluorescence analysis method for the detection of VB_12_ was established. The fluorescence suppression is attributed to inner filter effect because of the overlap between UV-vis absorption spectrum of VB_12_ and emission/excitation spectra of CDs. There is a good linear relation between VB_12_ concentration and (*F*_0_ − *F*)/*F*_0_ with the LOD is 93 nM. Compared with other method of fluorescence detection of VB_12_, this method is green environment and less pollution. The detection system is simple and fast, the linear range is wider and the sensitivity is higher.

## Conflicts of interest

There are no conflicts to declare.

## Supplementary Material

RA-008-C8RA03070G-s001
