# Supplementary material for: Carbon dots as fluorescent probes for detection of VB12 based on the inner filter effect
Source: RSC Adv. 2018 May 29;8(35):19786–90. doi: 10.1039/c8ra03070g (PMC9080768; doi:10.1039/c8ra03070g)
Supplement: RA-008-C8RA03070G-s001 [file RA-008-C8RA03070G-s001.pdf]

Electronic Supplementary Material (ESI) for RSC Advances.
This journal is © The Royal Society of Chemistry 2018

Electronic Supporting Information

Carbon Dots as Fluorescent Probe for Detection of VB_{12} Based on the Inner Filter Effect

X. Y. Sun*, M. J. Yuan, B. Liu and J. S. Shen

(College of Materials Science and Engineering, Huaqiao University, Key Laboratory of Molecular Designing and Green Conversions (Fujian University), 361021 Xiamen, China)

** E-mail: sunxy@hqu.edu.cn*

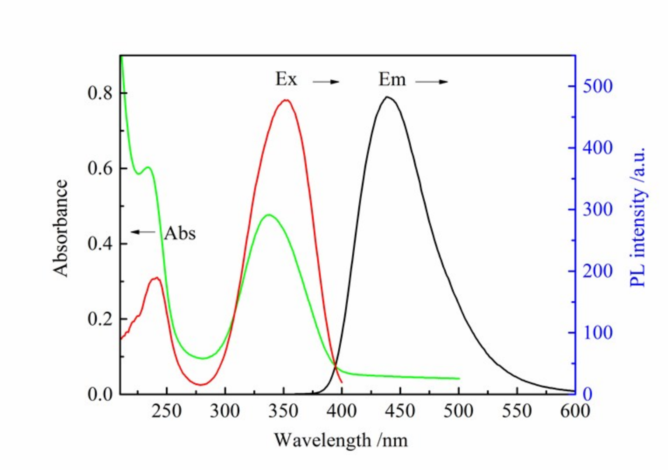

Figure S1 UV-vis absorption spectrum, PL excitation and PL emission spectra of CDs

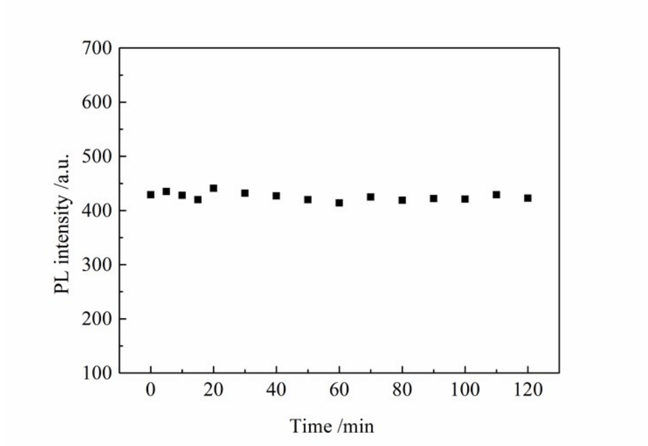

Figure S2 PL intensity of CDs versus irradiating time under UV lamp

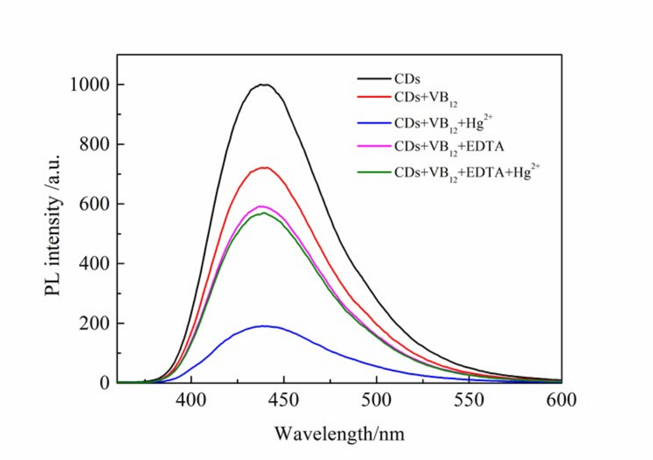

Figure S3 PL emission spectra of CDs under different conditions (Concentration of CDs was 1.2 μM, VB_{12} concentration was 8 μM, Hg^{2+} and EDTA concentration were 10 μM and 100 μM respectively)

Table S1 IFE of VB_{12} on the fluorescence of CDs

VB_{12}(μM)	*A*ex	*A*em	CF	*F*obsd	*F*cor	$F_{cor,o}/F_{cor}$
0	0.040	0.001	1.05	919.0	965.0	1
10	0.095	0.011	1.12	828.1	927.5	1.04
20	0.151	0.021	1.21	772.8	935.1	1.03
30	0.206	0.032	1.30	711.8	925.3	1.04
40	0.266	0.042	1.39	660.3	917.8	1.05
50	0.325	0.053	1.50	608.0	912.0	1.06
60	0.376	0.062	1.59	545.9	868.0	1.11
80	0.524	0.089	1.88	474.4	891.9	1.08
100	0.623	0.108	2.10	403.5	847.4	1.14
120	0.698	0.122	2.28	357.4	814.9	1.18
140	0.840	0.148	2.63	317.1	834.0	1.16
